# Current Status of Immunomodulatory and Cellular Therapies in Preclinical and Clinical Islet Transplantation

**DOI:** 10.1155/2011/637692

**Published:** 2011-10-20

**Authors:** Preeti Chhabra, Kenneth L. Brayman

**Affiliations:** ^1^Department of Surgery, University of Virginia, Charlottesville, VA 22908, USA; ^2^Division of Transplantation, Department of Surgery, University of Virginia, Charlottesville, VA 22908, USA; ^3^The Center for Cellular Transplantation and Therapeutics, University of Virginia, Charlottesville, VA 22908, USA

## Abstract

Clinical islet transplantation is a *β*-cell replacement strategy that represents a possible definitive intervention for patients with type 1 diabetes, offering substantial benefits in terms of lowering daily insulin requirements and reducing incidences of debilitating hypoglycemic episodes and unawareness. Despite impressive advances in this field, a limiting supply of islets, inadequate means for preventing islet rejection, and the deleterious diabetogenic and nephrotoxic side effects associated with chronic immunosuppressive therapy preclude its wide-spread applicability. Islet transplantation however allows a window of opportunity for attempting various therapeutic manipulations of islets prior to transplantation aimed at achieving superior transplant outcomes. In this paper, we will focus on the current status of various immunosuppressive and cellular therapies that promote graft function and survival in preclinical and clinical islet transplantation with special emphasis on the tolerance-inducing capacity of regulatory T cells as well as the *β*-cells regenerative capacity of stem cells.

## 1. Introduction

Type 1 diabetes (T1D) is a T-cell-mediated autoimmune disease that involves the progressive destruction of pancreatic *β* cells resulting in complete loss of insulin secretion [1]. While a combination of genetic predisposition and autoimmune processes contributes to its development, inflammatory mediators and innate immunity play a key role in the induction and amplification of the immune assault as well as in the maintenance of insulitis [2, 3]. Based on studies using animal models of autoimmune diabetes such as the biobreeding (BB) rat and the nonobese diabetic (NOD) mouse, the involvement of *β*-cell autoantigens, macrophages, dendritic cells (DCs), and T and B lymphocytes in the development of T1D has been established [4–6]. Antigen-presenting cells (APCs) such as macrophages and DCs are the first to infiltrate islets followed by CD4+ and CD8+ T lymphocytes, natural killer (NK) cells, and B lymphocytes. Macrophages play an essential role in the development and activation of cytotoxic CD8+ T cells, the final effectors of *β*-cell destruction [5]. Studies indicate that IL-12 secreted by macrophages may activate Th1-type CD4+ T cells and subsequently IL-2 as well as other proinflammatory cytokines released by the activated CD4+ T cells (for e.g., interferon-gamma IFN-*γ*, tumor necrosis factor TNF-*α* and TNF-*β*) contribute to maximizing the activation of cytotoxic CD8+ T cells. Activated CD4+ and CD8+ T cells act in unison to activate *β*-cell death via apoptosis. Additionally, IFN-*γ* released by CD4+ T cells may in turn activate macrophages to release proinflammatory cytokines and reactive oxygen species (ROS, e.g., superoxide, hydrogen peroxide, nitric oxide (NO), etc.). Ultimately, T-cell-mediated *β*-cell destruction is effected overall by the interplay between receptor-mediated interactions (e.g., Fas-Fas ligand, CD40 ligand, TNF-TNF receptor), secretion of proinflammatory cytokines and ROS as well as the release of granzymes and perforin from cytotoxic effector T cells. Furthermore, DNA damage in *β*-cells leads to the activation of poly (ADP-ribose) polymerase which rapidly depletes NAD that has been shown to be protective against radical-induced necrosis [7]. Interestingly, among the regulators of apoptosis, nuclear factor-kappa B (NF-*κ*B) has emerged as a master switch of cytokine-induced *β*-cell dysfunction and death [2]. Recognition of ligands by pattern-recognition receptors (PRRs, e.g., Toll-like receptor TLR3/4, RigI, MDA5) on *β*-cells leads to the activation of key transcription factors such as NF-*κ*B and signal transducer and activator of transcription 1 (STAT-1), inducing the release of chemokines and cytokines that recruit and activate immune cells, increase expression of MHC class I antigens, and activate proapoptotic signals resulting in *β*-cell destruction. Proinflammatory cytokines further induce STAT-1, NF-*κ*B, and interferon regulatory factor 3 (IRF-3) in *β*-cells, contributing to the maintenance and amplification of the immune processes that result in the progressive and selective destruction of pancreatic *β*-cells [2]. 

Several studies indicate the potential role of cytokines in modulating the immune response towards cytotoxic effector cell or suppressor cell dominance. T1D is thought to be a Th1-dominance disease with type 1 cytokines such as IFN-*γ*, TNF-*α*, IL-2, IL-12, and IL-18 actively involved in *β*-cell destruction. On the other hand, cytokines such as IL-4, IL-5, IL-10 as well as TGF-*β* secreted by Th2/Th3 cells are thought to inhibit *β*-cell destruction. Of tremendous interest is the recent discovery that Th17 cells are potent inducers of tissue inflammation and autoimmunity and may have a role in T1D as indicated in a study using NOD mice [8, 9]. A humanized T1D model has now been established in NOD-scid IL2R*γ* (null) mice to study the selective destruction of mouse islet *β*-cells by a human T-cell-mediated immune response, providing a valuable tool for translational research into T1D [10]. 

Data from large epidemiologic studies indicate that T1D accounts for 5% to 10% of the total cases of diabetes worldwide, with its incidence increasing by 2% to 5%. In the United States, the prevalence of T1D by 18 years of age is approximately 1 in 300 [11, 12]. Strategies to prevent or reverse T1D are broadly based on the concepts of *β*-cell regeneration, *β*-cell replacement, or protection of islets from immune destruction. While regenerative strategies broadly involve *β*-cell regeneration from progenitors/stem cells (e.g., putative *β*-cell progenitors/pancreatic stem cells; embryonic, mesenchymal, hematopoietic, and umbilical cord blood-derived stem cells (UCB-SCs), induced pluripotent stem cells (iPSCs), etc.) or neogenesis from ductal and non-*β*-cell progenitors, *β*-cell replacement strategies include transplantation of whole pancreas or islets of Langerhans or genetically engineered insulin-secreting cells. 

## 2. Islet Transplantation

The current standard of treatment for T1D requires lifelong exogenous insulin administration by either insulin pump or multiple daily injections which although successful by no means represents a cure, often resulting in hypoglycemic episodes and in microvascular complications in a high percentage of patients [13]. In the year 2000, islet transplantation was performed using the “Edmonton protocol” that consisted of induction therapy with a monoclonal antibody (mAb) against the interleukin-2 (IL-2) receptor (Daclizumab), and maintenance therapy with a calcineurin inhibitor (CNI, Tacrolimus) and a mammalian target of rapamycin (mTOR) inhibitor (Sirolimus). Over a followup period of approximately 11.9 months, all patients were insulin-free [14]. Subsequently, the reproducibility of this protocol was demonstrated by several studies. For instance, in 2006 an international multicenter trial demonstrated successful restoration of endogenous insulin producion and glycemic stability in 44% of transplant recipients at one year following the final islet transplantation. Persistent graft function defined by detectable C-peptide levels and improved glycemic control was observed in 70% of the recipients at the two-year followup although the insulin independence rate was disappointingly low (14%) [15]. The 2008 update from the Collaborative Islet Transplant Registry (CITR) summarizing the results from 279 recipients of one or more islet alone allotransplants between 1999 and 2007 also reported achievement of insulin independence in 23% of islet-alone recipients at 3-year after first infusion (insulin independence ≥2 weeks) while 29% were insulin dependent with detectable C-peptide, 26% had lost function, and 22% had missing data [16]. Furthermore, 70% comprising all those with one or multiple infusions achieved insulin independence at least once, of whom 71% were still insulin dependent 1 year later and 52% at 2 years translating to 50% achieving and retaining insulin independence for 1 year and 35% for 2 years for all islet-alone recipients. These results are consistent throughout the 8 years of followup included in the registry. A dramatic decrease in the prevalence of hypoglycemic episodes and substantial improvement in HbA1c levels was also observed. An increase from 2% preinfusion to 47%–69% at year 1 post-last infusion was observed in islet-alone recipients category with HbA1C less than 6.5% and absence of severe hypoglycemic episodes. Factors favoring the positive primary outcomes included amongst others a higher number of infusions, greater number of total islet equivalents infused, lower pretransplant HbA1c levels, related processing/infusion centers, islet viability more than 87%, larger islet size, and the use of protocols with daclizumab, etanercept or calcineurin inhibitors in the immunosuppressive regimens [16]. In fact, while successful islet transplantation outcomes have also been reported using islets isolated from a single donor [17, 18], Matsumoto et al. were the first to perform successful living donor islet transplantation to a recipient having brittle insulin-dependent diabetes with hypoglycemic unawareness [19]. Results obtained from recipients of autologous islet transplantation after pancreatectomy at the University of Minnesotta demonstrated a remarkably limited rate of decline of insulin independence despite infusion of a lower *β*-cell mass [20]. Of 173 islet autotransplant recipients, insulin independence was achieved in 32% within the first year. Of those with insulin independence, 74% remained insulin independent at the 2-year followup, 46% at 5 years and 28% at 10 years. These results are remarkably higher than the CITR data and highlight the strong impact of autoimmune and alloimmune injury on graft survival in type 1 diabetics receiving an infusion of allogeneic islets [16, 20, 21]. The Edmonton protocol can also be applied to patients undergoing either islet after kidney (IAK) or simultaneous islet kidney (SIK) procedures resulting in a high rate of graft function and insulin independence, although the morbidity is higher when compared to patients undergoing solitary islet transplantation for brittle T1D [22]. In fact, an improvement in cardiovascular function for up to 3 years of follow-up was observed in type 1 diabetic patients with end-stage renal disease (ESRD) receiving an IAK transplant compared with the kidney-only group. Also improvements in kidney graft function, survival rates, and stabilization of microalbuminuria among uremic patients [23] as well as an improvement in vascular diabetic complications [24] were observed following IAK transplantations. Currently, several Phase I and Phase II clinical trials to study IAK in T1D are ongoing (ClinicalTrials.gov Identifier: NCT00708604; NCT00888628; NCT01123187; NCT00468117). Although restoration of endocrine function and glucose homeostasis can be achieved by transplantation of either whole pancreas or islets of Langerhans, each procedure has distinct advantages over the other. While whole pancreas transplantation has been shown to be very effective at maintaining an euglycemic state over a sustained period of time providing the graft recipient an opportunity to benefit from the improvement of blood glucose control, it is associated with a significant risk of surgical and postoperative complications [25]. Islet transplantation on the other hand, offers substantial benefits in terms of being minimally invasive, reducing incidences of debilitating hypoglycemic episodes/hypoglycemic unawareness, lowering daily insulin requirements, improving levels of glycated hemoglobin, and affording potential insulin independence [26–28]. Furthermore, it allows a window of opportunity for attempting various therapeutic manipulations of islets prior to transplantation aimed at achieving superior transplant outcomes. However, despite the impressive advances in this field, a limiting supply of islets, inadequate means for preventing islet rejection and the deleterious diabetogenic and nephrotoxic side effects associated with chronic immunosuppressive therapy preclude its wide-spread applicability. Also, long-term insulin independence in islet transplant recipients is frequently lost by the fifth year of followup [29]. Nonetheless, the overall positive impact of islet transplantation on metabolic control in T1D continues to spur efforts worldwide to develop new strategies directed at achieving long-lasting insulin independence following islet transplantation. 

### 2.1. Immune Strategies That Promote Engraftment and Function of Transplanted Islets

Current challenges to successful transplantation include amongst others (a) the loss of functional islet mass resulting from nonspecific inflammatory responses as well as from preexisting and/or transplant-induced, autoimmune-mediated islet destruction and alloimmune rejection and (b) the failure of the newly transplanted islets to revascularize successfully resulting in inefficient engraftment and primary dysfunction of islets. Herein, we will review some of the various preclinical and clinical efforts to protect islet grafts from the deleterious effects of innate and adaptive allo- and autospecific immune responses as well as to promote vasculogenesis, thereby increasing longitudinal graft survival and function. These strategies include amongst others immunomodulatory therapies and immunosuppressive regimens. Immunomodulatory therapies (e.g., monoclonal antibody therapies, costimulatory signaling blockade, IL-1 receptor antagonist therapy, cellular therapies, etc.) act by (a) providing immunoregulatory cytokines such as IL-4, IL-10, or TGF-*β* or (b) by inhibiting proinflammatory cytokines or (c) by altering the balance between TH1 and TH2 cells or (d) by protecting islets from immune destruction by encapsulation and so forth. Immunosuppressive regimens on the other hand act by either (a) suppressing the innate and adaptive immune response through various strategies that include binding to specific cytoplasmic proteins that inhibit IL-2 secretion and subsequent T-cell expansion (CNIs Cyclosporine and Tacrolimus) or (b) by suppressing IL-2R signaling thereby inactivating T cells (Sirolimus) or (c) by inhibiting cell division of lymphocytes (Azathioprine) [30]. Currently, combinatorial therapies that promote vasculogenesis/angiogenesis of islets and islet regeneration/*β*-cell expansion and that target antigen specific/nonspecific and antibody-specific immunomodulations that readjust the underlying immunologic imbalance to stop/reverse the *β*-cell-specific immune destruction and maintain immune tolerance together with steroid-sparing or -free immunosuppressive regimens lacking nephrotoxicity or diabetogenicity are of immense therapeutic value towards generating successful islet transplantation outcomes. 

#### 2.1.1. Blocking the Instant Blood Mediated Inflammatory Reaction: Pharmacologic and Encapsulation Strategies

 One of the first inflammatory assaults encountered by the islets on contact with ABO-compatible blood following transplantation via hepatic portal vein is the instant blood-mediated inflammatory reaction (IBMIR) [31, 32]. IBMIR is a thrombotic reaction characterized by activation of both the coagulation and complement cascades that results in the disruption of islet morphology and function, posing a serious obstacle to successful islet engraftment. Activation of the complement and the generation of anaphylatoxins C3a and C5a leads to recruitment of polymorphonuclear (PMN) leukocytes, upregulation of adhesion molecules on the endothelium and platelets, generation of ROS, and induction of cytokine release [33, 34]. Islets themselves secrete both tissue factors (TFs) that trigger IBMIR [35] and chemokines including monocyte chemoattractant protein 1 (MCP1), chemokine C-X-C motif ligand 8(CXCL8), and chemokine C-C motif ligand 2 (CCL2) that might participate in recruitment of PMNs and monocytes/macrophages to the site of engraftment [36–39]. In fact, a significant association between TF and CCL2 released *in vitro* by islets and biochemical indices of coagulation was observed in patients after islet transplantation [36] indicating that reduction of the islet proinflammatory state may be a means to reduce the early posttransplant complications and possibly improve islet engraftment. To this end, strategies attempted for improving *β*-cell graft survival include the use of OptiPrep gradient media during purification of human islets. This media has been shown to significantly reduce cytokine/chemokine production including IL-1*β*, TNF-*α*, IFN-*γ*, IL-6, IL-8, MIP-1*β*, MCP-1, and RANTES during the 48-hour culture after isolation thereby improving *β*-cell survival during pretransplant culture [40]. Similarly, embedding mouse islets in reconstituted basement membrane extract (BME) for 24 or 48 hours partially protected islets from anoikis by decreasing caspase-3 levels and increasing *α*3 integrin, focal adhesion kinase (FAK) protein level, and FAK activity suggesting a beneficial role in preservation of viability and function of isolated islets. Furthermore, expression of transcription factor pancreatic and duodenal homeobox 1 (PDX-1) was preserved at 48 hours indicating a positive contribution of BME to cell activity [41]. 

Pioneering work has been done by the group of Nilsson and Korsgren in developing clinically applicable strategies to inhibit the activation of the coagulation and complement systems and the infiltration of leukocytes into islets that characterize the IBMIR. For instance, they demonstrated the effectiveness of low MW-dextran sulphate in inhibiting IBMIR in a nonhuman primate model of pancreatic islet transplantation, providing the basis for a protocol that can be used to abrogate the IBMIR in pig-human clinical islet xenotransplantation [32]. As a result of their studies, a clinical trial to assess the safety and efficacy of LMW-sulfated dextran on posttransplant islet function in people with T1D is ongoing (ClinicalTrials.gov Identifier: NCT00790439). They also developed a biotin/avidin technique to conjugate preformed heparin complexes to the surface of islets that protected against intraportal thrombosis and inhibited the IBMIR without increasing the bleeding risk or inducing acute or chronic toxicity in the islets [35]. On a similar principle, they demonstrated that human aortic endothelial cell- (EC-) coated islets reduced activation of coagulation and complement, platelet/leukocyte consumption in blood as well as infiltration of CD11b+ cells [42, 43]. Furthermore, addition of mesenchymal stem cells (MSCs) to this composite indicated enhanced EC proliferation, sprout formation, and ingrowth of ECs into the islets demonstrating the clinical potential of composite EC-MSC islets in improving islet angiogenesis, revascularization, and immune regulation [44]. Optimizing the EC-islet coculture methods to enable human EC coating of pig islets would offer new strategies to improve xenogenic islet transplantation outcomes. Recently, immobilization of human soluble complement receptor 1 (sCR1) on the islet cell surface through poly(ethylene glycol)-conjugated phospholipid (PEG-lipid) was shown to effectively inhibit complement activation and protect islets against attack by xenoreactive antibodies and complement without loss of islet cell viability or insulin secretion ability, demonstrating an efficient means to control early islet loss in clinical islet transplantation [45]. Coating islet surfaces with PEG-lipid followed by further modification with a fibrinolytic enzyme urokinase further increased the efficacy of this procedure [46, 47]. These reports highlight the potential of tissue-targeted chemistry to reduce donor-cell-mediated procoagulant and proinflammatory responses. The anaphylatoxin C5a elicits a broad range of proinflammatory effects and most likely plays a crucial role in IBMIR. In a recent mouse intraportal islet transplant study, Satomi's group demonstrated that C5a inhibitory peptide (C5aIP) significantly suppressed the thrombin-antithrombin complex formation, improving both curative rate and glucose tolerance as well as significantly suppressing the expression of TF on granulocytes in recipient livers [48]. Suppression of TF expression attenuated cross-talk between the complement and coagulation cascades leading to improvement in islet engraftment indicating that C5aIP therapy in combination with conventional anticoagulants could represent a beneficial intervention in clinical islet transplantation. Similar reduction in the expression of TF and MCP-1 in human islets was demonstrated by the addition of nicotinamide to the culture medium in an *in vitro* loop model [49]. Surface reengineering of pancreatic islets with recombinant azido-thrombomodulin also resulted in a significant reduction in islet-mediated thrombogenicity [50]. Nilsson's group had also demonstrated that administration of a specific thrombin inhibitor Melagatran significantly reduced IBMIR *in vitro* in a dose-dependent manner [51] as did sCR1/heparin therapy [52, 53] or TP10/immune-suppressive drug treatment following intraportal transplantation of porcine islets to cynomlogus monkeys [54]. Other anticoagulant or complement inhibitors to block IBMIR in preclinical studies include N-acetyl L-cysteine that efficiently inhibited the procoagulant activity of human recombinant TF in human islet cell preparations at clinically relevant concentrations without cellular toxicity [55]. 

Fetal and neonatal porcine islets express a cell surface antigen containing the epitope—Gal*α*1—3Gal*β*1—4GlcNAc-R (“*α*-gal”) to which humans have complement fixing antibodies that cause immediate rejection of transplanted islets [56–59]. This response may be prevented by (a) inhibition of the functional “*α*-gal” activity using knockout [60] or knockin procedures involving human alpha (1,2)-fucosyltransferase [61] or N-acetyl-glucosaminyl transferase III gene expression [62] or (b) by depletion of anti-pig antibodies or complement from serum or (c) the use of GT knockout (*α*1,3-galactosyltransferase gene knockout [GTKO]) pigs [63] as the sources of islets. Expression of thrombomodulin [64], TF pathway inhibitor [65], CD39 the endothelial ecto-nucleoside triphosphate diphosphohydrolase, and haem oxygenase also demonstrate potential value for coagulation control in pig-to-human xenotransplantation. Prevention of coagulation dysfunction [66] or expression of human complement-regulatory proteins such as CD46 (membrane cofactor protein) [67, 68], human decay accelerating factor (DAF, CD55) [64, 69], CD59 [70–72] or all three [73]; or expression of anticoagulant and antiplatelet molecules within the graft may also afford some protection [57]. For instance, adenovirus-mediated expression of the human complement regulatory proteins DAF (CD55) or CD59 protected adult porcine islets from complement-mediated cell lysis by human serum [74] as did a low-molecular mass factor VIIa (FVIIa) inhibitor that indirectly blocked both membrane bound and alternatively spliced forms of TF *in vitro* [75]. Recombinant antithrombin III may also ameliorate both early graft damage and the development of systemic coagulation disorders in pig-to-human xenotransplantation [76]. These strategies in parallel with physical methods such as encapsulation may contribute significantly in reducing the thrombogenicity of pig islet xenografts. 

Several intravascular and extravascular devices involving encapsulation technology to overcome destruction of the graft by immune cells and large antibodies have been described. While intravascular macrocapsules are anastomosed to the vascular system as AV shunt, extravascular macrocapsules are mostly diffusion chambers transplanted at different sites and extravascular microcapsules are transplanted in the peritoneal cavity. The major advantage of the intravascular device is that direct contact with the blood stream ensures ample oxygen and nutrient supply enhancing graft survival and function. Use of vascularized bioartificial devices was among the few cases that obtained long-term allo- and xenogeneic islet survival in totally pancreatectomized dogs [77, 78]. However, thrombosis is a major challenge, requiring intense anticoagulation therapy. The extravascular devices on the other hand offer minimal surgical risk, transplantation at extrahepatic sites, lack of thrombosis, and ease of retrieval from recipient in case of pericapsular fibrotic overgrowth. Both these devices however remain vulnerable to small molecules such as cytokines/chemokines and nitric oxide as well as hypoxic stress. Coencapsulation of islets with various agents such as erythrocytes [79] and sertoli cells [80] that release immunosuppressive factors or with factors that enhance revascularization such as vascular endothelial growth factor (VEGF) [81], photosynthetic oxygen generator algae [82] and so forth or with factors like bioengineered insulin-like growth factor-II (IGF-II) [83] that promote pancreatic *β*-cell survival have been shown to improve insulin secretion and glycemic control posttransplantation. Naturally obtained alginate hydrogels are most widely used in islet transplantation with barium alginate demonstrating long-term immunoprotection in both allo- and xenotransplantation settings. For instance, a significant decrease in the secretion of MCP1 and improvement in the islet activity was observed when SD rat islets were microencapsulated with novel sulfated glucomannan barium alginate (SGA) and allotransplanted intraperitoneally into diabetic Lewis rats [84]. Similar immunoprotection was observed when islets co-encapsulated with angiogenic protein in permselective multilayer alginate-poly-L-ornithine-alginate microcapsules were transplanted into an omentum pouch [85]. An extravascular (subcutaneous) transplant macrochamber called the “*β*Air” device, consisting of islets immobilized in a thin alginate-impregnated, hydrophilized Teflon membrane and simultaneously supplied with oxygen by daily refueling with oxygen-CO_2_ mixture provided immunoprotection and sustained islet viability and function following allogeneic subcutaneous transplantation into healthy minipigs [86]. Transplantation of porcine islets microencapsulated in highly purified barium alginate into streptozotocin- (STZ-) induced diabetic Wistar rats resulted in long-term normoglycemia without immunosuppression [87]. Thus, by providing a solution to the hurdles of immunological rejection and the risk of infection with porcine pathogens in clinical xeno-islet transplantation [88], the use of encapsulation devices may help to circumvent the shortage of allogeneic human donor organs [56]. Alginate macroencapsulation of pig islets was also shown to correct STZ-induced diabetes in primates up to 6 months without immunosuppression [89]. In a pilot clinical trial wherein microencapsulated human islets were transplanted into the central abdominal region of non-immunosuppressed patients with T1D, improvement in glycated hemoglobin and the disappearance of hypoglycemia was observed, although the patients remained on insulin therapy [90]. In a very recent clinical study, Elliot et al. demonstrated transitory insulin independence of several months duration using the microencapsulation technique [56]. Interestingly, the treatment appeared to significantly decrease severe hypoglycemic episodes and reduce/abolish hypoglycemic unawareness episodes, even in the absence of insulin independence. Evidence of xenosis in the xenotransplants recipients though diligently sought could not be found.

#### 2.1.2. Immunotherapeutic Strategies Targeting the Non-Antigen Specific Immune Response

Approximately 60% of transplanted pancreatic islet tissue undergoes apoptosis within the first several days in experimental models of syngeneic islet transplantation [13, 91, 92]. Infiltration of graft by PMNs and tissue macrophages and elevated levels of proinflammatory cytokines following islet transplantation strongly suggest the involvement of nonspecific innate inflammatory events in mediating cellular injury. Other mediators involved in the early loss of pancreatic islets include NO, prostaglandins (PGs), and reactive oxygen intermediates (ROIs) [92, 93]. Several studies indicate that inhibition of non specific inflammation improves the function and survival of islet grafts. For instance, Yasunami's group demonstrated that V-alpha14 NKT cell-triggered IFN-*γ* production by Gr-1+CD11b+ cells mediated early graft loss of syngeneic transplanted islets [94]. Adenosine administration suppressed NKT cell-mediated IFN-*γ* production of neutrophils in the livers of graft-recipient mice, leading to prevention of early loss of transplanted syngeneic and allogeneic islets. Similar results were observed in a recent study wherein recipients receiving ATL therapy (ATL146e or ATL313) achieved normoglycemia more rapidly than untreated recipients following syngeneic islet transplantation indicating improved survival and functional engraftment of transplanted marginal mass of islets [91]. Histological examination of grafts suggested reduced cellular necrosis, fibrosis, and lymphocyte infiltration in agonist-treated animals. Administration of adenosine A(2A) receptor agonists also improved *in vitro* glucose-stimulated insulin secretion (GSIS) by an effect on leukocytes, suggesting a potentially significant interventional strategy for reducing inflammatory islet loss in clinical transplantation. A similar beneficial effect associated with reduction in inflammatory cell infiltration and *β*-cell death by apoptosis was observed following administration of diannexin [95]. Sequential combination of a JNK inhibitor SP600125 and nicotinamide plus simvastatin also protected porcine islets from peritransplant apoptosis and inflammation [96]. This combination therapy increased *β*-cell viability index and viability of porcine islets cultured overnight as well as significantly increased the islet survival rate *in vivo*. Intraductal administration of JNK inhibitor significantly suppressed mRNA expression levels of IL-1*β*, IFN-*γ*, TNF-*α*, IL-6, IL-8, and MCP-1 *in vivo* and also decreased the concentration of IL-1*β* and IL-8 in culture supernatant *in vitro* possibly representing an alternative target for suppression of porcine islet inflammation. Very recently, administration of bilirubin too was shown to reduce the serum levels of inflammatory mediators including IL-1*β*, TNF-*α*, soluble intercellular adhesion molecule 1 (ICAM1), MCP-1 and NO, inhibit the infiltration of Kupffer cells into islet grafts, restore insulin-producing ability of transplanted islets and enhance glucose tolerance in diabetic mouse recipients [97]. IL-1*β* plays a key role in causing pancreatic islet dysfunction and apoptosis [92]. Through a cascade of intracellular events, IL-1*β* secreted by neutrophils and macrophages was shown to down-regulate GLUT2, up-regulate inducible NO synthase (iNOS) and cyclooxygenase-2, and activate NF-*κ*B which in turn mediated the transcription of a multitude of genes, including IL-1, IL-6, TNF-*α*, ICAM-1, VCAM-1, ELAM-1, iNOS, PGE2, COX-2, and EP3 [92, 97–99]. The inhibition of IL-1*β* induced COX-2 and EP3 gene expression by sodium salicylate [99] as well as of iNOS and COX-2 tyrosine kinase were all shown to enhance pancreatic isle *β*-cell function [100]. Also deficiency of NOX2 decreased ROI/proinflammatory cytokine production and *β*-cell apoptosis, thereby protecting against STZ-induced *β*-cell destruction and development of diabetes in mice [101].

**Inhibition of Toll-Like Receptor (TLR) Signaling**

A family of 10 functional, human, innate, immune signaling receptors called TLRs plays an important role in the host's innate defense as well as adaptive immunity. Recognition of conserved pathogen-associated microbial patterns (PRRs) stimulates specific TLRs on APCs and T lymphocytes to induce proinflammatory cytokines, chemokines, and interferons. The endogenous TLR ligands include amongst others peptidoglycan, extradomain A of fibronectin, lipopolysaccharide, heparan sulfate, hyaluronan, high-mobility group box protein 1 (HMGB1), Hsp60, exogenous products such as bacterial lipoprotein, CpG DNA, flagellin, and double stranded viral RNA [102–105]. Murine islets constitutively express TLR2 and TLR4, and TLR activation upregulates intra-islet production of cytokines and chemokines. A recent study indicated that TLR2 and TLR4 signaling could initiate islet graft failure and that HMGB1 was an early mediator [106]. Following transplantation into STZ-induced diabetic syngeneic mice, islets exposed to LPS or peptidoglycan had primary graft failure caused by recipient CD8+ T cells with intra- and peri-islet mononuclear cell inflammation. NF*κ*B activation in stressed islets was prevented in the absence of both TLR2 and TLR4. Transplantation of TLR2/4(−/−) islets reduced proinflammatory cytokine production and improved islet survival. Yet another study indicated that early intraportal islet graft failure in mice was associated with increased proinflammatory cytokines, HMGB1 expression, NF*κ*B activation, caspase-3, and TUNEL-positive cells [107]. Deficiency of TLR4 in donor, but not in recipient, inhibited NF*κ*B activation, reduced proinflammatory cytokines and improved viability of islet grafts. Blockade of HMGB1 with anti-HMGB1 monoclonal antibody (mAb, 2 g7) inhibited inflammatory reactions as evidenced by reduced TNF*α* and IL-1ss production and improved islet viability. Thus, these results indicate that inhibition of TLR4 activation represents a novel strategy to attenuate early graft failure following intraportal islet transplantation. Matsuoka et al. demonstrated that mice HMGB1 receptors TLR2 or receptor for advanced glycation end products (RAGE) but not TLR4, failed to exhibit early islet graft loss [108]. Mechanistically, HMGB1 stimulated hepatic mononuclear cells (MNCs) *in vivo* and *in vitro*, upregulated CD40 expression and enhanced IL-12 production by DCs, leading to NKT cell activation and subsequent NKT cell-dependent augmented IFN-*γ* production by Gr-1+CD11b+ cells. Treatment with either IL12- or CD40L-specific antibodies prevented early islet graft loss. Also, treatment with a HMGB1-specific antibody inhibited IFN-*γ* production by NKT cells and Gr-1+CD11b+ cells following intraportal islet transplantation, indicating that the HMGB1-mediated pathway was a potential interventional target for improving the efficiency of islet transplantation.

**Protection by Suppressor of Cytokine Signaling (SOCS)**

The protective effect of suppressor of cytokine signalling (SOCS)-3 in mouse and rat islets subjected to cytokine stimulation has been characterized by R*ø*nn et al. [109]. Using mice with *β*-cell-specific Socs3 expression as well as a Socs3-encoding adenovirus construct, a significant resistance to cytokine-induced apoptosis and impaired insulin release was demonstrated by the transgenic islets. GSIS, insulin content or glucose oxidation were not affected by SOCS3. Rat islet cultures transduced with Socs3-adenovirus too displayed reduced cytokine-induced NO and apoptosis associated with inhibition of the IL1-induced NF*κ*B and mitogen-activated protein kinase (MAPK) pathways. While transplanted Socs3 transgenic islets were not protected in diabetic NOD mice, they did show a prolonged graft survival when transplanted into diabetic allogeneic BALB/c mice indicating that SOCS3 may represent a target for pharmacological or genetic engineering in islet transplantation for treatment of T1D. A more recent study using chimeric adenovirus vector (Ad5F35-SOCS1) to enhance SOCS1 expression in isolated Sprague-Dawley rat islets indicated decreased levels of active caspase 3 and intranuclear apoptosis inducing factor (AIF) after treatment with TNF-*α* and cyclohexamide *in vitro *[110]. Caspase 3 is the central executioner caspase activated by upstream cascades in a caspase-dependent apoptosis pathway while AIF is a key mitochondrial protein that translocates to the nucleus in a caspase-independent apoptosis pathway. Transplantation of Ad5F35-SOCS1-infected islets into STZ-induced diabetic recipients resulted in significantly prolonged functional graft survival. Also, decreased caspase 3 activation and AIF translocation to nucleus was observed in Ad5F35-SOCS1-infected islet grafts in the early posttransplant period indicating that SOCS1 mediated protection of islet grafts from apoptosis through caspase-3-dependent and AIF-caspase-independent pathways [110]. These results were supported by another study in which transgene expression of SOCS-1 rendered islets significantly more resistant to cytokine-induced cell death after treatment with TNF-*α* alone and in combination with IFN-*γ* [111]. Furthermore this resistance could be correlated with significant inhibition of the transcription factor interferon regulatory factor-1 (IRF-1), reflecting enhanced cell survival signals as well as impairment of IFN*γ*-induced class I MHC upregulation [93, 111]. Importantly, SOCS1-Tg islets significantly reversed STZ-induced diabetes indicating that intragraft expression of SOCS1 rendered islets insensitive to the deleterious effects of cytokines; a finding of potential significance in the development of therapies against acute allograft rejection [111].

**Protection by X-linked Inhibitor of Apoptosis Proteins (XIAPs)**

Adenovirally delivered, transient overexpression of X-linked inhibitor of apoptosis proteins (XIAPs) in a growth-regulatable *β*-cell line (*β*TC-Tet) indicated that *in vitro*, XIAP-expressing *β*TC-Tet cells were markedly resistant to apoptosis in an ischemia-reperfusion injury model system as well as following exposure to cytokines [112]. Subcutaneous transplantation of these Ad-XIAP transduced *β*TC-Tet cells into immunodeficient mice resulted in reversal of diabetes in 3 days as versus 21 days for Ad-*β*Gal transduced control cells. These results were recapitulated in a recent study wherein XIAP overexpression inhibited *β*-cell apoptosis in syngeneic islet transplants, reducing the number of islets as well as decreasing the number of days required to restore euglycemia, thereby raising the possibility that *ex vivo* XIAP gene transfer in islets prior to transplantation had the potential to increase donor islet mass available for transplantation by allowing more efficient use of the limited existing supply of human islets thereby enhancing graft function and long-term transplant success [113]. In comparison with this adenoviral delivered gene transfer approach, a “*nonviral*” approach was recently described involving the transfection of IDO cDNA– containing plasmids into rat islets using Lipofectamine. Indoleamine 2,3-dioxygenase (IDO) is an enzyme that plays a critical role in suppressing T-cell responses inducing fetomaternal tolerance [114]. Allotransplantation of IDO expressing islets into STZ-induced diabetic Lewis rats was shown to reverse diabetes and maintain glucose metabolic control. Also, survival of IDO-transfected islet allografts transplanted without any immunosuppression was superior to that observed in diabetic rats receiving nontransfected islets.

**Protective Effect of Haem Oxygenase-1 (Ho-1, Hmox1)**

Overexpression of the cytoprotective protein haem oxygenase-1 (HO-1) has been shown to reduce the deleterious effects of cytokine-induced apoptosis and oxidative stress in transplantable islets [115–118]. For instance, HO-1 upregulation by protoporphyrins (Cobalt-protoporphyrin CoPP, Ferrous-protoporphyrin FePP), powerful inducers of the HO-1, in a murine insulinomae *β*-TC3 cell line and in freshly isolated murine islets was shown to exert a protective effect from apoptosis induced *in vitro* with proinflammatory cytokines [115, 116] or Fas engagement [117], while *in vivo* HO-1 upregulation resulted in improved islet function in a syngeneic model of marginal mass islet transplantation in rodents [118]. These results were supported by a similar study in which CoPP treatment of donor mice for the induction of HO-1 beginning one-day prior to islet isolation plus a 9-day posttransplantation course resulted in enhanced engraftment of syngeneic islets and improved blood glucose levels and glycemic control [119]. Short-course peritransplant administration of CoPP also led to long-term DBA/2 islet allograft survival in a sizable proportion of C57BL/6 mice recipients [120]. Furthermore, preconditioning of islets with FePP alone led to improved graft survival in untreated recipients and further increased the proportion of long-term surviving grafts in CoPP-treated recipients. Preconditioning also resulted in reduction of class II expression. Peritransplant administration of protoporphyrins to allograft recipients also resulted in transient powerful immunosuppression with reduced lymphocyte proliferative responses, increased proportion of regulatory T cells (CD4+CD25+) and decreased mononuclear cell infiltrating the graft, paralleled by a systemic upregulation of HO-1 expression, probably contributing to the induction of donor-specific hyporesponsiveness in a proportion of the protoporphyrin-treated animals. The transgenic expression of haem oxygenase-1 in pancreatic *β*-cells of NOD mice prolonged graft survival and afforded protection from autoimmune damage [121]. Reduced levels of proinflammatory cytokines/chemokines, proapoptotic gene expression, and amounts of ROS/RNS from islets were observed, with islets more resistant to TNF*α*- and IFN*γ*-induced apoptosis, providing valuable insight into the development of better strategies for clinical islet transplantation in patients with T1D.

**Protective Regulatory Role of Interferon Regulatory Factor 1 (IRF1)**

A key role of IRF1 in immune-mediated *β*-cell destruction has been indicated [93]. IRF-1 is a downstream target of IFN-*β*/signal transducer and an activator of STAT-1. Deletion of IRF1 in islets was associated with a higher prevalence of primary nonfunction, reduced insulin secretion and shorter functioning graft survival. Cytokine-exposed Irf1 (−/−) islets and INS1E cells transfected with Irf1 siRNA showed increased expression of Mcp1 (Ccl2), Ip10 (Cxcl10), Mip3*α* (Ccl20), and Inos (Nos2) mRNA and elevated production of MCP-1 and nitrite compared with controls. *In vivo*, Irf1 (−/−) islets displayed a higher potential to attract immune cells, reflected by more aggressive immune infiltration in the grafted islets. IL-1 receptor antagonist partly restored the cytokine-induced secretory defect *in vitro* and completely prevented primary non function *in vivo*. These data indicate a key regulatory role for IRF1 in insulin and chemokine secretion by pancreatic islets under inflammatory attack.

**Beneficial Effect of Redox Modulation Strategies**

*β*-cells are especially vulnerable to free radical and inflammatory damage due to reduced antioxidant defenses. A recent study was done to determine the efficacy and benefit of a redox modulation strategy using the catalytic antioxidant (CA) FBC-007 in improving islet preservation. The results indicated that incubation of human islets with FBC-007 before syngeneic, suboptimal syngeneic, or xenogeneic transplant protected islets from STZ-induced damage and significantly increased their function [122]. Diabetic murine recipients of catalytic antioxidant-treated allogeneic islets exhibited improved glycemic control posttransplant and demonstrated a delay in allograft rejection. Systemic administration of catalytic antioxidant to recipients further delayed allograft rejection suggesting that addition of a redox modulation strategy would be a beneficial clinical approach for islet preservation in syngeneic, allogeneic, and xenogeneic transplantation.

#### 2.1.3. Targeting Adaptive Immunity: The Effectiveness of Immunosuppressive and Tolerance-Inducing Immunotherapeutic Strategies

Presentation of alloantigens in combination with costimulatory molecules by APCs activates signal transduction pathways, including the calcium-calcineurin pathway, triggering the T-cell response [21]. Subsequent release of IL-2 amongst other molecules, activates the mTOR pathway initiating cell proliferation and hugely expanding the number of effector T cells. Activated B lymphocytes too produce alloantibodies against donor HLA antigens.

**Costimulatory Signaling Blockade**

Several studies indicate that blockade of costimulatory signal pathways in experimental transplantation models by cytotoxic T lymphocyte antigen-4 immunoglobulin (CTLA4Ig) or CD40LIg enhances islet graft survival [123, 124]. For instance, human islets infected with AdCTLA4Ig-IRES2-CD40LIg that simultaneously expressed CTLA4Ig and CD40LIg proteins significantly prolonged graft survival of murine islet xenografts, downregulated expressions of Th1-cells-related cytokines, and inhibited inflammatory cell infiltration [125]. Caspase inhibitor therapy (EP1013) in combination with costimulation blockade (CTLA4-Ig) too prevented engraftment phase islet loss and markedly reduced islet mass required to reverse diabetes [126]. Furthermore, EP1013/CLTA4-Ig cotherapy significantly increased graft survival by reducing the frequency of alloreactive IFN-*γ* secreting T cells and increasing the frequency of intragraft FoxP3+ Treg cells. The results of these studies indicate that by minimizing immune stimulation and reducing the requirement for long-term immunosuppressive therapy, these combination therapies have tremendous potential in improving clinical transplantation.

Simultaneous blockade of CD40/CD154 and ICAM/lymphocyte function-associated antigen (LFA)-1 also prolonged allograft survival [127]. Larsen's group targeted adhesion molecule LFA-1 that is preferentially expressed on donor-specific memory T (TM) cells and has been implicated in costimulation blockade-resistant transplant rejection [128]. Short-term induction therapy with the LFA-1-specific antibody TS-1/22 in combination with either Basiliximab (an IL-2R*α*-specific mAb) and Sirolimus or Belatacept (a high-affinity variant of the CD28 costimulation-blocker CTLA4Ig) prolonged islet allograft survival in nonhuman primates by masking LFA-1 on TM cells. Inhibition of the generation of alloproliferative and cytokine-producing effector T cells expressing high levels of LFA-1 *in vitro *was also observed. Neutralization of costimulation blockade-resistant populations of T cells with LFA-1-specific induction therapy has tremendous potential in transplantation. The efficacy of Efalizumab, a blocking monoclonal antibody directed at LFA1, as part of maintenance therapy regimen with Sirolimus was being tested (ClinicalTrial.gov identifier NCT00672204). Efalizumab is no longer available for clinical use. An anti-coinhibitory receptor B and T lymphocyte attenuator monoclonal antibody (anti BTLA, PJ196) has been reported to prolong fully MHC-mismatched cardiac allograft survival [129]. A study testing the synergistic effect of anti-BTLA monoclonal antibody PJ196 and CTLA4Ig costimulatory blockade in islet allotransplantation showed that downregulation of BTLA on the surface of lymphocytes along with accumulation of cells with regulatory phenotype at the graft site promoted islet allograft acceptance indicating that this combination may prove to be an effective adjunctive strategy for inducing long-term allograft survival. Similarly, co-stimulatory signal blocker LEA29Y (Belatacept) has shown promising data in transplantation studies in primates [130]. Clinical trials using LEA29Y in islet transplantation are currently underway, for example Clinical Islet Transplantation consortium's clinical trial CIT-04 is designed to study islet transplantation in type 1 diabetes with LEA29Y (belatacept) maintenance therapy (University of Alberta, Emory University) and another ongoing Phase II interventional study (ClinicalTrials.gov Identifier: NCT00501709) is using Belatacept for the prevention of autoimmune destruction and rejection of human pancreatic islets following transplantation for T1D. Apart from costimulation blockade of the CD28/CD80/CD86 and CD40/CD154 pathways as a means of inducing peripheral tolerance, several new costimulatory molecules have been identified in recent years including OX40 that belongs to the TNF receptor family and is expressed on activated T cells [131]. OX40 ligation with OX40L enhances cytokine production, proliferation, and survival [131]. Mechanistic studies indicate that anti-OX40L, treatment preserves Treg numbers and OX40 blockade offers better xenoislet graft survival than CTLA4Ig in the spontaneous autoimmune NOD model, offering a novel therapeutic target for xenoislet graft protection in type 1 diabetic patients [132]. Recently, a combination therapy consisting of anti-CD40L, anti-OX40L, and anti-CD122 mAbs prolonged islet allograft survival in alloantigen-primed mice [133]. This combination of mAbs also inhibited accelerated rejection mediated by donor reactive memory T cells, known to accelerate allograft rejection, in xenoantigen-primed mice cells by inhibiting cellular and humoral immune responses.

**Monoclonal Antibodies**

Anti-CD3 antibody has been shown to induce tolerance in allograft transplantation [134], reverse autoimmunity in NOD mice [135] and slow the progression to permanent diabetes in humans with recent-onset diabetes [136]. In female NOD mice, oral anti-CD3 mAb was effective in reversing diabetes, allowing pregnancies and extending longevity [135]. Treatment of diabetic transgenic mice (NOD background) expressing the human *ε* chain of the CD3 complex with Otelixizumab (an anti-human CD3 antibody) resulted in durable disease remission dependent on transferable T-cell-mediated tolerance [137]. In single-donor islet transplantation studies [138], anti-CD3 mAb [hOKT3c1(Ala–Ala)] contributed to promising results [139]. A Phase II clinical trial studying the efficacy of anti-CD3 mAb in the treatment of recent onset T1D (ClinicalTrial.gov identifier NCT00378508) is currently underway. Another trial studying the efficacy of hOKT3*γ*1 (Ala-Ala) combined with Sirolimus and delayed Tacrolimus in promoting islet allograft survival has just been completed. In an adoptive transfer model, the use of anti-CD134L mAb was shown to effectively prevent activation of CD4+ memory T cells and significantly prolong islet survival, similar to the manner anti-CD122 mAb prevents activation of CD8+ memory T cells [140]. Short-term administrations of a combination of anti-LFA-1 and anti-CD154 monoclonal antibodies too induced tolerance to neonatal porcine islet xenografts in mice [141]. The use of anti-CD134L and anti-CD122 mAbs in addition to co-stimulatory blockade with anti-CD154 and anti-LFA-1 prolonged secondary allograft survival in an alloantigen-primed model and significantly reduced the proportion of memory T cells [142]. It also increased the proportion of Tregs in the spleen, inhibited lymphocyte infiltration in the graft, and suppressed alloresponse of recipient splenic T cells suggesting that the combination therapy of four mAbs could significantly suppress the function of memory T cells and prolong allograft survival in alloantigen primed animals. Similar results were obtained by the same group using a combination of CTLA4Ig, antiCD40L, anti-LFA-1, and anti-OX40L while another study used a combination of anti-CD40L, anti-OX40L, and anti-CD122 mAbs antibodies to inhibit accelerated rejection mediated by memory T cells in xenoantigen-primed mice [143].

**IL-21-Targeted Therapy**

The efficacy of an IL-21-targeted therapy (IL-21R/Fc, an IL-21-neutralizing chimeric protein) on prevention of diabetes in NOD mice, in combination with syngeneic islet transplantation has recently been investigated [144]. Results showed that IL-21-responsiveness by CD8+ T cells was sufficient to mediate islet allograft rejection and that combining neutralization of IL-21 with islet transplantation restored glucose homeostasis and resulted in recovery from autoimmune diabetes. Since the absence of IL-21 signaling prevented islet allograft rejection indicating a robust influence of IL-21 on a graft-mounted immune response, these findings imply that therapeutic manipulation of IL-21 may serve as a suitable treatment for patients with T1D.

**DNA Vaccination Strategy**

A therapeutic DNA vaccination strategy for autoimmunity and transplantation has also recently been described wherein intradermal injection of plasmid DNA encoding glutamic acid decarboxylase (GAD) polypeptide, which is synthesized in both pancreatic islet and skin tissue, ameliorated new-onset T1D in NOD mice and increased skin allograft survival in a BALB/c-C57BL/6 model system in a donor-specific manner [145]. Furthermore, only CpG-methylation of plasmid DNA coding for GAD was required in order to significantly increase skin allograft survival after immunization of recipient; codelivery of a cDNA coding for the proapoptotic BAX protein, which was shown previously to be essential for successful therapy of autoimmune diabetes in NOD mice by induction of FoxP3+ regulatory T cells, was not necessary. This study therefore revealed the promising potential for autoimmunity-targeting DNA vaccination to be applied to clinical transplantation.

**Localized Immunosuppression Using Glucocorticoid Microspheres**

The ability of poly(D,L-lactic) acid (PLA) and poly(D,L-lactic glycolic acid (PLGA) microspheres of the soft corticosteroid loteprednol etabonate (LE) loaded within a biohybrid device to provide localized immunosuppression and reduce systemic side effects over an extended period has been evaluated in a set of early exploratory experiments with diabetic rats receiving islet transplantation. While sustained release of microspheres at low concentration showed no cytotoxicity on the viability of the MIN-6 insuloma cell line *in vitro*, animals treated using a biohybrid device loaded with microspheres showed improved results compared to those treated by delivery in solution form with an osmotic minipump [146]. Sustained local delivery formulation for glucocorticoids dexamethasone phosphate and LE using PLA microspheres showed promise in their ability *in vivo *to prolong allograft survival in rats after tapering of systemic immunosuppression, compared to control groups [147]. Also, doses delivered locally were approximately hundredfold smaller than those typically used in systemic treatments.

**Induction of Tolerance by Regulatory T cells (Tregs)**

Donor alloantigen-specific CD4+CD25+ Tregs play an important role in inducing and maintaining tolerance to donor alloantigens *in vivo*. By actively modulating or suppressing the deleterious CD4+ and CD8+ T-cells-mediated immune response to donor alloantigens, Tregs may prevent rejection and mediate linked unresponsiveness [148]. *In vivo*, these cells are dependent on interleukin IL-10 and CTLA4 for functional activity. As part of clinical tolerance strategies, the immunosuppressive ability of peripheral T-cell-depleting agents such as anti-CD52 mAb Alemtuzumab (Campath-1H) and antithymocyte globulin (ATG) have been tested in humans with autoimmunity and in transplantation scenarios [139, 149]. Lopez et al. were the first to report that ATG but not Alemtuzumab or the IL-2R antagonists caused rapid and sustained expansion of CD4+CD25+ T cells in cocultures with human peripheral blood lymphocytes and displayed enhanced expression of the regulatory markers glucocorticoid-induced TNF receptor, CTLA4 and FoxP3 as well as efficient suppression of a direct alloimmune response of the original responder lymphocytes [150]. The induction of Tregs depended on the production of Th2 cytokines in the generating cultures. This study demonstrated the therapeutic potential of ATG in promoting the generation of Tregs for cellular therapy in autoimmunity and clinical transplantation. Recently, a clinically relevant immunoregulatory strategy based on treatment of NOD mice with murine Thymoglobulin (mATG) and CTLA4-Ig to prevent allo- and autoimmune activation in a stringent model of islet transplantation and diabetes reversal was investigated. The results revealed that transplant recipients experienced a complete abrogation of autoimmune responses and significant downregulation of alloimmunity in response to treatment [151]. Furthermore, this striking effect was confirmed by 100% diabetes reversal in newly hyperglycemic NOD mice and 100% indefinite graft survival of syngeneic islet transplantation (NOD*scid* into NOD mice). An induction immunosuppressive therapy regimen consisting of rabbit ATG and the monoclonal antibody to CD20 rituximab (Rituxan) promoted long-term islet allograft survival in cynomolgus macaques maintained on rapamycin monotherapy [152]. Several clinical trials aimed at studying the safety and effectiveness of islet transplantation combined with various immunosuppressive regimens that include ATG for treatment of type 1 diabetic individuals experiencing hypoglycemia unawareness and severe hypoglycemic episodes are either completed or underway. These include amongst others an islet after kidney transplantation trial with ATG and etanercept (ClinicalTrials.gov Identifier: NCT00468117) and islet transplantation trials with ATG, Everolimus and cyclosporine (ClinicalTrials.gov Identifier: NCT00286624); with Rituxan (Rituximab), Thymoglobulin (ATG), Zenapax (Daclizumab) and Rapamune (Sirolimus) (ClinicalTrials.gov Identifier: NCT00468442); with Lisofylline, ATG, basiliximab, Sirolimus and Tacrolimus, (ClinicalTrials.gov Identifier: NCT00464555); and with Raptiva, ATG, and sirolimus (ClinicalTrials.gov Identifier: NCT00672204) amongst others. In a recent study, patients receiving intraportal allogeneic islet transplants were maintained on immunosuppression consisting of ATG induction and maintenance with sirolimus or mycophenolate mofetil (MMF) and costimulation blocker belatacept or the anti-LFA-1 antibody Efalizumab which permit long-term islet allograft survival [153]. The results indicated that both regimens were effective and well tolerated, with the calcineurin inhibitor/steroid-sparing islet protocols resulting in long-term insulin independence and belatacept proving to be an effective alternative in improving graft function and longevity while minimizing renal and *β*-cell toxicity. Because Tregs strongly suppress the immune response in syngeneic islet transplantation and improve graft survival and function, several approaches are now emerging to induce/increase host Tregs activity in the transplant setting, including amongst others, systemic TGF-*β*1 therapy [154]. A recent study demonstrated that islet-specific Treg induced from the BDC-6.9 TCR transgenic mouse by activation of T cells in the presence of TGF-*β* could suppress both spontaneous diabetes as well as transfer of diabetes into NOD*scid* mice by diabetic NOD spleen cells or activated BDC-2.5 TCR transgenic Th1 effector T cells [155]. In the latter transfer model, the authors demonstrated that infiltration of Tregs into pancreas caused a reduction in the number of effector Th1 T cells and macrophages, and also inhibited effector T-cell cytokine and chemokine production. Transfection of effector T cells with a dominant-negative TGF-*β* receptor demonstrated that *in vivo* suppression of diabetes by TGF-*β*-induced Treg is TGF-*β* dependent.

**Rapamycin**

Rapamycin is a key component of the immunosuppressive regimen in clinical islet transplantation. The impact of rapamycin, on human islet engraftment and function was assessed in 10 patients with T1D before islet transplantation [156]. The results indicated that pretreatment with rapamycin was associated with a reduction in chemokines CCL2 and CCL3 pretransplantation and a dampened chemokine response post-transplantation, potentially improving clinical islet engraftment by an anti-inflammatory mechanism. In rat-to-mouse islet xenotransplantation, administration of anti-CD154 mAb and rapamycin induced Treg-mediated tolerance [157]. Rapamycin has been shown to allow expansion, proliferation, and regulatory function of both murine and human naturally occurring CD4+CD25+FOXP3+ Tregs (nTregs), which are pivotal for the induction and maintenance of peripheral tolerance [158]. Pothoven et al. demonstrated that rapamycin-conditioned Balb/c donor bone-marrow-derived DCs (BMDCs) had significantly enhanced ability to induce CD4+CD25+FOXP3+ iTregs of recipient origin (C57BL/6 (B6)) *in vitro* under Treg driving conditions compared to unmodified BMDCs [154]. These *in vitro* induced CD4+CD25+FOXP3+ iTregs exerted donor-specific suppression *in vitro* and prolonged allogeneic islet graft survival *in vivo* in RAG(−/−) hosts upon coadoptive transfer with T-effector cells. The CD4+CD25+FOXP3+ iTregs also expanded and preferentially maintained FOXP3 expression in the graft draining lymph nodes and were able to induce endogenous naïve T cells to convert to CD4+CD25+FOXP3+ T cells. Thus, rapamycin-conditioned donor BMDCs can be exploited for *in vitro* differentiation into donor antigen-specific CD4+CD25+FOXP3+ iTregs capable of effectively controlling allogeneic islet graft rejection. Strom's group demonstrated a triple therapy approach that combined administration of rapamycin and agonist IL-2- and antagonist IL-15-related cytolytic fusion proteins [159]. This treatment promoted very long-term engraftment/tolerance of allogeneic islets in both spontaneously diabetic NOD mice and IL-2-deficient recipients by limiting the early expansion of activated T cells, preserving and even exaggerating their subsequent apoptotic clearance, and further amplifying the depletion of these activated T cells by antibody-dependent mechanisms, while preserving CD4+CD25+ T-cell-dependent immunoregulatory networks. In T1D, Treg activity has been demonstrated in the pancreatic lymph node, but the *in vivo* activity of Tregs during suppression in pancreas remains poorly characterized. A clinical study wherein nTreg numbers and function were examined in a unique set of patients with T1D who underwent rapamycin monotherapy before islet transplantation indicated that while rapamycin monotherapy did not alter the frequency and functional features, namely, proliferation, and cytokine production of circulating nTregs, it increased their capacity to suppress proliferation of CD4+CD25− effector T-cells [160]. These findings demonstrate that rapamycin directly affects human nTreg function *in vivo*, by refitting their suppressive activity, whereas it does not directly change effector T-cell function.

**Induction of Tolerance Through Suppression of TLRs**

TLR2-induced IL-6 secretion from APCs has been shown to reverse the suppressive function of Tregs [104, 161] and in combination with TGF*β*-induced Th17 cells resulted in enhanced inflammation [103, 104, 162, 163] and prevention of transplantation tolerance [163]. In a very recent study to understand the mechanism by which different inflammatory signals affect transplantation tolerance and immunity, it was determined that TLR2 ligand peptidoglycan inhibited FOXP3 expression in both natural Tregs (nTregs) and TGF*β*-driven adaptive Tregs (aTregs) independent of paracrine Th1, Th2, and Th17 cytokines [104]. While TLR2-induced inhibition of FOXP3 was independent of STAT1, STAT3, STAT4, and STAT6, it was dependent on Myd88 and IRF. Binding of induced IRF1 to IRF1 response elements (IRF-E) in the FOXP3 promoter and intronic enhancers negatively regulated FOXP3 expression to suppress Treg function. Furthermore by inducing divergent chromatin changes at the FOXP3 locus, inflammatory IL-6 and TLR2 signals regulated Treg suppressor function and reduced graft survival in an islet transplantation model. Schroppel's group demonstrated that deficiency of TLR4 in islet graft recipients prolonged graft survival. Low dose rapamycin-treatment of TLR4(−/−) recipients induced permanent and prolonged engraftment of 45% of the islet graft that was dependent on the presence of CD4+CD25+FOXP3+ Tregs [164]. Naïve CD4+CD25− T cells cultured with the TLR4 ligand lipopolysaccharide showed enhanced IL-4, IL-6, IL-17, and IFN-*γ* secretion and inhibited TGF*β*-induced FOXP3+Treg generation. These results indicated that inhibition of recipient TLR4 activation at the time of transplantation decreased proinflammatory signals and allowed for Treg generation. Approaches such as generating TLR agonists/antagonists, creating monoclonal antibody to TLRs, blocking key molecules in the signaling pathways and downmodulating TLR signaling may be of immense benefit in the treatment of T1D and islet transplantation.

**Other Tolerance Inducing Treatments**

Interestingly, treatment of islet graft recipients with bilirubin prolonged islet allograft survival via a Treg-dependent manner in which CD4+CD25+ Treg cells were necessary for tolerance induction and graft acceptance. Bilirubin treatment also promoted *de novo* generation of Tregs possibly accounting for the observed protective effects [165]. A combination treatment of Hmox1 induction, carbon monoxide, and bilirubin administration led to long-term survival and tolerance toward islet allografts by promoting FOXP3+ Tregs and inducing and maintaining tolerance in the recipient [166]. VAG539 is a water soluble derivate of VAF347, a low-molecular-weight compound that activates the aryl hydrocarbon receptor (AhR). Oral administration of VAG539 promotes long-term graft acceptance and active tolerance in Balb/c mice that receive MHC-mismatched pancreatic islet allograft, resulting in increased frequency of splenic CD4+CD25+FOXP3+ T cells *in vivo* and improved CD4+CD25+FOXP3+ T-cell survival *in vitro *[167]. Interestingly, transfer of CD11c+ DCs but not of CD4+ T or CD19+ B cells, from VAG539-treated long-term tolerant hosts into mice that recently underwent transplantation resulted in donor (C57Bl/6)-specific graft acceptance and in a significantly higher frequency of splenic CD4+CD25+FOXP3+ Tregs. Furthermore, the transfer of these CD4+CD25+ Tregs into recently transplanted mice promoted islet graft acceptance. Also, cell therapy with *in vitro* VAF347-treated bone-marrow-derived mature DCs prevented islet graft rejection, and reduced OVA-specific T-cell responses in OVA-immunized mice. Taken together these data indicate that activation of AhR induces islet allograft-specific tolerance through direct as well as DC-mediated effects on Treg survival and function. Transient depletion of dividing T cells by administration of ganciclovir for 14 days, induced at the time of allogeneic islets transplantation into diabetic transgenic mice that express a thymidine kinase (TK) conditional suicide gene in T cells also resulted in allograft tolerance in 63% of treated mice accompanied by a 2- to 3-fold persistent increase in the proportion of CD4+CD25+FOXP3+ Treg within 3 weeks only in allograft-bearing mice. Additionally, lymphocytes from tolerant mice could transfer tolerance to naïve allografted recipients [168]. Similar results were obtained after cytostatic hydroxyurea treatment in normal mice suggesting that the transient depletion of dividing T cells represented a novel means of immunointervention based on disturbance of T-cell homeostasis and subsequent increase in Treg proportion.

**In Vivo and In Vitro Expansion of Tregs**

Nagahama et al. established a protocol for *in vivo* and *in vitro* alloantigen-specific expansion of naturally arising CD4+CD25+ regulatory T-cells (Treg) to establish antigen-specific dominant tolerance to allogeneic transplants [169]. They showed that *in vivo* exposure of CD4+CD25+ T cells from normal naive mice to alloantigen in a T-cell-deficient environment elicited the spontaneous expansion of alloantigen-specific CD4+CD25+ nTregs capable of suppressing allograft rejection mediated by subsequently transferred naive T cells, leading to long-term graft tolerance. Similarly they demonstrated that antigen-specific expansion of nTregs can be achieved *in vitro* by stimulating CD4+CD25+ T cells from normal animals with alloantigen in the presence of high doses of IL-2. The expanded Tregs were even capable of suppressing secondary mixed leukocyte reaction *in vitro* and, following adoptive transfer, were able to establish antigen-specific long-term graft tolerance. Francis et al. have recently demonstrated that graft-protective Treg arise *in vivo* both from naturally occurring FOXP3+CD4+ Tregs and from non-regulatory FOXP3−CD4+ cells [170]. Interestingly, the induction of tolerance also inhibited CD4+ effector cell priming with T cells from tolerant mice demonstrating impaired effector function *in vitro*. Thus, by converting potential effector cells into graft-protective Tregs and by expanding alloreactive naturally occurring Tregs, adaptive tolerance was induced. Adoptive cell therapy using patient-specific CD4+CD25+ Tregs as individualised medicine to promote clinical transplantation tolerance is very promising [171]. If this principle is to be applied to clinical tolerance induction, strategies targeting potential effector cells will have to be investigated for successful generation of alloreactive Tregs that may be critical for long-term allograft survival without chronic immunosuppression.

**The Role of Tolerance-Inducing Dendritic Cell Therapies in Treg and TH17 Cells Interconversion**

Activated CD4+ T cells develop into Th1, Th2, or Th17 subsets based on the cytokines they produce and distinct effector functions. Th17 cells produce IL-17A, IL-17F, Il-10, IL-22, and IL-21 and play a role in host defense against infections, and in inducing tissue inflammation in autoimmune disease [172]. The broad distribution profile of IL-17 and IL-22 receptors guarantees induction of a massive tissue reaction. The involvement of differentiation factors (TGF-*β* plus IL-6 or IL-21), the growth and stabilization factor (IL-23), and the transcription factors (STAT3, ROR*γ*, and ROR*α*) in the development and stabilization of Th17 cells has recently been identified [173]. Th17-derived IL-21 plays an important role in the amplification of Th17 cells [8]. Based on the evidence of immunosuppressive TGF-*β* participation in the differentiation of Th17 cells, the Th17 lineage appears to have a close relationship with CD4+CD25+FOXP3+ Tregs. Th17 cells were shown to be involved in islet transplant rejection, and blockade of IL-23R was shown to positively correlate with reduction in IL-17 expression in a dose-dependent manner [174]. While the combination of anti-CD154 mAb and IL-23R antibody was shown to prevent the acute rejection to some extent, no significant difference was observed when compared with the anti-CD154mAb alone. RelB(lo) DCs generated in the presence of an NF-*κ*B inhibitor induce Tregs and suppress inflammation. A very important recent study showed that while tolerizing RelB(lo) DCs were able to significantly inhibit diabetes progression when administered to 4-week-old NOD mice, IL-1*β* produced in response to islet autoantigen presentation reduced the immunosuppressive capacity of Treg cells and promoted their conversion to Th17 cells [175]. RelB(lo) DCs exacerbated the IL-1-dependent decline in Treg function and promoted Th17 conversion. This study highlights the importance to entertain caution while using tolerizing DC therapies that regulate islet autoantigen priming and prevent diabetes and that progression past the IL-1*β*/IL-17 checkpoint signals the need for adapting other tolerizing strategies.

**Current Combinatorial Therapeutic Strategies in Clinical Islet Transplantation**

Currently, islet transplantation followed by ATG/alemtuzumab (Campath-1H, monoclonal). Anti-CD52 Ab/hOKT3*γ*/anti-CD25 (daclizumab) induction therapy along with a sirolimus-based, prednisone-free maintenance regimen in combination with MMF and low Tacrolimus as well as drugs that demonstrate powerful immunosuppressive/anti-inflammatory potency in the absence of nephrotoxicity and diabetogenicity are under investigation [153, 176–179]. Other drugs currently in Phases II or III of development include Otelixizumab (anti-CD3), Teplizumab (anti-CD3), Rituximab (anti-CD20), Abatacept (CTLA4Ig), DiapPep 277 (heat shock protein), and GAD and Oral Insulin amongst others. GLP-1R agonists like exendin-4 stimulate *β*-cell proliferation and neogenesis and inhibit *β*-cell apoptosis while DPPIV inhibitors increase cell insulin content and are therefore of immense benefit in the above mentioned combination therapies for preserving and expanding *β*-cell mass following transplantation. The activity of potent proinflammatory TNF*α* can be inhibited by etanercept, a recombinant TNF*α* receptor protein. A high success rate of insulin independence was achieved using a protocol in which Etanercept was administered as induction therapy following single donor islet transplantation, in combination with Prednisone, Daclizumab, and rabbit ATG [180–182]. Combined treatment with Etanercept and Exenatide in addition to the Edmonton immunosuppressive protocol was shown to reduce the number of islets needed to achieve insulin independence [183] and improve glucose control and graft survival in patients who needed a second transplantation because of progressive graft dysfunction [183, 184]. These combinatorial strategies could also include encapsulation of islets with nanofiber scaffolds or biomatrices or permselective alginate microcapsules synthesized to release immunosuppressive drugs or drugs that stimulate vasculogenesis/angiogenesis to improve trasnplantation outcomes at extrahepatic sites [185].

#### 2.1.4. Role of Immunomodulatory Stem Cells in Promoting Graft Survival

Several studies indicate that nonimmunogenic multipotent MSCs apart from playing an important role in *β*-cell replacement therapies owing to their versatile differentiation and *ex vivo* expansion potential, are also cytoprotective immune modulators, exerting therapeutic effects by promoting graft protection, tissue revascularization, and *β*-cell survival in islet transplantation [186]. Bone-marrow-derived MSCs (BM-MSCs) can enhance repair and regeneration, not only by repopulating damaged tissue, but also by reducing inflammation. Also, they allow transplantation across MHC barriers since they do not possess cell surface human leukocyte antigen (HLA) or MHC class II molecules. Kim et al. evaluated the therapeutic potential of autologous MSCs in preventing graft rejection following allogeneic rat islet transplantation and demonstrated that when combined with cyclosporine A therapy, graft survival attained more than 100 days in 33% of autologous MSCs-plus-CsA-treated recipients [187]. Splenocytes from autologous MSC-plus-CsA-treated rats exhibited a reduced MLR proliferative response to donor stimulators and increased IL-10 release. Interestingly, IL-10 induced by CD11b+ cells and IL-10 activated Tregs played a role in MSC-mediated immune modulation in the rat islet allograft. The authors demonstrated that autologous MSCs-plus-CsA downregulated immune responses and induced donor-specific T-cell hyporesponsiveness by reducing the production of proinflammatory cytokines and inducing anti-inflammatory cytokine production, especially that of IL-10, during the early post-transplantation period. Tregs contributed at a later phase. Thus, the combined use of autologous MSCs and low-dose CsA exerted a synergistic immunosuppressive effect in an islet allograft model. These results were supported by the demonstration that triple-dose administration of either syngeneic or allogeneic MSCs was able to prevent acute rejection and improve glycemic control in diabetic rats receiving marginal islet mass transplantation via the portal vein [188]. Reduced levels of pro-inflammatory cytokines and glucose as well as low-grade rejections were observed up to 15 days after transplantation indicating the ability of MSCs to prolong graft function by preventing acute rejection. Also the efficacy of MSCs was independent of the administration route and comparable to that of immunosuppressive therapy indicating that MSCs may play an important role in preventing acute rejection and improving graft function in portal vein pancreatic islet transplantation. It is also noteworthy to mention that xenotransplantation of three-dimensional spheroid bodies (SBs) formed under special induction conditions from endometrial mesenchymal stem-like cells (SB-EMSCs) into immunocompromised mice with STZ-induced diabetes restored blood insulin levels to control values and greatly prolonged the survival of graft cells. These results suggest that EMSCs not only played a novel role in the differentiation of pancreatic progenitors, but could also functionally enhance insulin production to restore the regulation of blood glucose levels. It will be interesting to dissect the role of these cells in immunomodulation and vasculogenesis in an *in vivo* transplantation model [189]. 

 The pancreatic endocrine potential of hESC-derived CD34+ cells has recently been demonstrated by Goodrich et al. who transplanted sheep with these cells by *in utero* intraperitoneal injections prior to development of the immune system in the fetus so that tolerance toward foreign antigens was acquired during gestation and persisted in the adult [190]. In animals transplanted with differentiated cell populations and followed up to 55 months after transplantation, they detected human DNA and insulin messenger RNA in sheep pancreases as well as human C-peptide in serum. As few as 23,500 cells were able to achieve long-term sustainable *β*-cell-like activity. These results along with the absence of teratomas, combined with the hematopoietic potential of these cells suggest that not only do hESC-derived CD34+ cells have potential for long-term *in vivo* endocrine cellular activity but could also be used for the induction of immunological tolerance and bone marrow chimerism prior to cellular therapy for diabetes. A phase II islet transplantation clinical trial at the University of Miami is currently ongoing using monoclonal antibody Campath-1H for induction of immunosuppression combined with the simultaneous infusion of islets with donor CD34+ enriched bone marrow cells (ClinicalTrials.gov identifier: NCT00315614) to reverse hyperglycemia, induce a state of donor-specific tolerance and eliminate the need for continuous immunosuppressive therapy. A study to investigate the immunomodulatory role of mobilized autologous hematopoietic stem cells (HSCs) via antagonism of the CXCR4-CXL12 axis in promoting islet engraftment following allotransplantation demonstrated mobilization of HSCs and prolongation of islet graft survival that was further enhanced by the addition of rapamycin to anti-CXCR4 therapy, inducing a robust and transferable host hyporesponsiveness. Mobilized HSCs expressed high levels of the negative costimulatory molecule programmed death ligand 1 (PDL1) and suppressed the *in vitro* alloimmune response. Thus, targeting the CXCR4-CXCL12 axis mobilized autologous HSCs and promoted long-term survival of islet allografts via a PD-L1-mediated mechanism. Halting the CXCR4 antagonist-mediated HSC release by administration of an ACK2 (anti-CD117) mAb restored allograft rejection [191]. 

The innate anti-inflammatory and immunosuppressive potential of human amniotic epithelial cells (AECs) to create localised immuneprivilege in an *in vitro* islet cell culture system as an alternative to immunosuppressive drug therapy was investigated. Islets transduced with bioengineered cellular constructs composed of human islets and AEC (islet:AEC) demonstrated sustained, physiologically appropriate insulin secretion and reduced mitogen-induced PBL proliferation suggesting that transplanted islets may benefit from the immune-privilege status conferred on them as a consequence of their close proximity to human AEC and that this approach may reduce the need for chronic systemic immunosuppression [192].

### 2.2. Enhancing Graft Survival by Promoting Vasculogenesis and Reducing Hypoxia

In experimental islet transplantations, both blood perfusion as well as the tissue oxygen tension of the grafted islets are chronically decreased in the transplanted islets, indicating that reestablishment of an appropriate microvascular supply is an essential prerequisite for successful islet engraftment. The islet grafts depend upon endothelial cells and microvessels in the implantation organ for derivation of a new vascular system [193, 194]. Improved islet graft survival and function have been observed on exposure to growth factors such as basic fibroblast growth factor (bFGF), endothelial cell growth factor *α* and particularly VEGF, which has been known to contribute significantly to the vascularization of transplanted islets [195]. In humans, the VEGF family of homodimeric glycoproteins consists of VEGF-A, -B, -C, -D, and placental growth factor [194]. Interestingly, although pancreatic islets continuously express VEGF-A [196], upon subjection to hypoxia following transplantation devascularized grafted islets significantly increase their expression of VEGF, initiating revascularization and maintaining the vascular permeability [196–199]. VEGF-A stimulates EC permeability and chemotaxis through cognate VEGF receptors and is a prerequisite for islet endothelial fenestration [194–200]. Korsgren and Magnusson's group showed that immobilizing heparin on the islet surface was useful in achieving complete coverage of islets with VEGF-A as a means of attracting ECs to induce angiogenesis and revascularization, ultimately improving islet revascularization and engraftment in pancreatic islet transplantation [35, 44]. A very recent study indicates that concomitant transplantation of isolated islets with ECs can prolong islet graft survival in diabetic rats [201]. Cartilage oligomeric matrix protein-angiopoientin-1 (COMP-Ang1) is a specific growth factor that induces vascularization via the Tie2 or Tie1 receptor. Using an *in vitro* angiogenesis assay based on a three-dimensional collagen-based culture system, Park et al. recently demonstrated that the transduction of COMP-Ang1 into islets significantly increased angiogenesis [202]. COMP-Ang1 transduced islets also attenuated hyperglycemia in syngeneic STZ-induced diabetic C57BL/6 mice and enhanced glucose tolerance. In another study, following subcutaneous transplantation of BALB/c islets in VEGF and hepatocyte-growth-factor (HGF) supplemented matrigel basement membrane matrix into diabetic *scid* mice [203], histopathologic analysis of the functioning grafts harvested at 15 days revealed significantly increased blood vessel formation and increased number of islets. Enhanced intercellular adhesion molecule (ICAM) and vascular cell adhesion molecule (VCAM) within the islets was also observed suggesting stable blood vessel formation. Transcription factors focal adhesion kinase phosphorylation and extracellular signal-regulated kinase1/2 phosphorylation were also increased (8-fold and 4.6-fold, respectively). These results suggest synergistic enhancement of angiogenesis by VEGF and HGF following islet transplantation resulted in stable engraftment. In order to drive delivery of growth factors such as VEGF and FGF into the dense islet interior, Chow et al. developed heparin-binding peptide amphiphile (HBPA)/heparin nanofiber gels that can activate heparin-binding, angiogenic growth factors [204]. Infiltration of bioactive nanofibers in the interior of islets acted as an artificial extracellular matrix (ECM) improving cell viability and function and enhancing their vascularization in the presence of growth factors such as FGF2 and VEGF. The intraislet nanofibers helped retain FGF2 within the islet for 48 h and increased cell viability significantly for at least 7 days in culture. Furthermore, enhanced insulin secretion was observed with the nanofibers for 3 days in culture. Delivery of FGF2 and VEGF in conjunction with the HBPA/heparin nanofibers also induced a significant amount of islet EC sprouting from the islets into a peptide amphiphile 3D matrix. This approach may have a significant impact on islet transplantation. Mahato coexpressed human VEGF (hVEGF) and human IL-1 receptor antagonist (hIL-1Ra) as well as human HGF (hHGF) and hIL-1Ra in human islets using Adv-hVEGF-hIL-1Ra Adv-hHGF-hIL-1Ra constructs to study their effect on *β*-cell proliferation and revascularization of islets [205]. A dose- and time-dependent expression of hVEGF and hIL-1Ra or hHGF and hIL-1Ra by islets was observed that led to a decrease in caspase-3 activity and apoptosis induced by a cocktail of TNF-*α*, IL-1*β*, and IFN-*γ*. Also, transduction of islets with these bipartite Adv vectors prior to transplantation in diabetic NOD*scid* mice reduced blood glucose levels and increased serum insulin and c-peptide levels. Immunohistochemical staining of the graft revealed positivity for human insulin, hVEGF or hHGF, and von Willebrand factor. Transduction with Adv-caspase-3-shRNA also prevented islets from cytokine-induced apoptosis and improved islet transplantation [205]. 

The therapeutic potential of hyperbaric oxygen therapy (HBO) in reducing hypoxia, enhancing vessel maturation, and improving engraftment of intraportal islet transplants by promoting angiogenesis in the critical period following transplantation has also been demonstrated [206]. In this study, hyperbaric oxygenation combined with implantation of a foam dressing, vacuum-assisted wound closure (foam+VAC) to create a prevascularized site was used to achieve better results in microencapsulated xenogeneic cell transplantation [206, 207]. Interestingly, following intraportal islet transplantation of porcine islets to diabetic NMRI nu/nu mice, the combination of cytokines with hypoxia resulted in a strong induction of cell death that could be blocked dose-dependently by a selective IKK-*β* inhibitor that caused systemic NF-*κ*B inhibition, significantly prolonging islet graft survival [208]. Under hypoxia, NF-*κ*B activity impaired expression of antiapoptotic genes BCL-xL, c-FLIP and survivin. NF-*κ*B thus appeared to have an antiapoptotic role under normoxia, while low oxygen conditions decreased its activity and transformed it to a proapoptotic transcription factor in pancreatic islets suggesting that NF-*κ*B inhibition represented a potential strategy to improve islet transplantation efficiency. Significant islet loss related to reduced survival of large islets compromised by hypoxia has been observed under standard culture conditions. In order to improve the islet graft quality prior to transplantation, rat islets have been cultured for 48 h in a liquid-liquid interface culture system (LICS) using perfluorodecalin, a method of culture that avoids exposure of islets to relative hypoxia [209]. Results indicated that this protocol optimised culture conditions, which preserved both islet viability and significantly increased their ability to engraft successfully after intraportal transplantation and could be used for islet transportation. Evaluating neovascularization and correlating angiogenesis with metabolic and functional islet graft condition in diabetic mice is a crucial aspect in assessing graft survival. To this end, very recent studies demonstrated the successful use of dynamic contrast-enhanced magnetic resonance imaging (DCE-MRI) after intravenous injection of gadolinium [210, 211]. MRI has also been used to effectively image isolated mouse islets labeled with novel MRI contrast agent, chitosan-coated superparamagnetic iron oxide (CSPIO) nanoparticles as long as 18 weeks after transplantation as well as islets labeled with Feridex-polyethyleneimine complex [212, 213]. A recent study using a dual-purpose therapy/imaging probe consisting of therapeutic (siRNA targeting apoptosis-related gene human caspase-3) and imaging (magnetic iron oxide nanoparticles, MN) moieties showed that treatment with the probe resulted in significantly better survival of transplanted islets that could be monitored by *in vivo* magnetic resonance imaging (MRI) [214].

#### 2.2.1. Stem Cells in Promoting Angiogenesis/Vasculogenesis

Bone-Marrow-derived stem cells (BMSCs) have been shown to promote islet graft function and survival by initiating angiogenesis [215, 216]. A recent study involving cotransplantation of bone marrow cells with islets was associated with enhanced islet graft vascularization and function. A significant increase in new peri-islet vessels [216] as well as staining for VEGF was observed. The presence of pancreatic duodenal homeobox-1 (Pdx-1) was detected in BMSCs with an increase in staining over time. Protein array measurements conducted in human islets cocultured with whole human BM for approx. 7 months indicated upregulated levels of angiogenesis factors VEGF-a, PDGF, KGF, TIMP-1, and angiogenin as well as lower protein levels of angiopoietin-2 [215]. Depletion of VEGF-a, eKGF, and PDGF significantly reduced islet vascularization. BM-induced vascularization showed significant EC distribution and islet vascularization was linked to islet growth. Furthermore a 28.66-fold increase in insulin and 24.4-fold glucagon gene expression was also observed. These data indicate that BMSCs induced endocrine cell regeneration via regulation of angiogenesis factors. Transplantation of genetically marked whole bone marrow from Tie2-Cre/ZEG mice into lethally irradiated wild-type mice evoked pronounced proliferation of recipient ECs while significantly increasing *β*-cell mass and reducing the hyperglycemia of mice subjected to *β*-cell damage by STZ [217]. A study indicated that bone marrow cells produced nerve growth factor (NGF) and promoted angiogenesis around transplanted islets [218]. Biochemical and histological analyses following syngeneic cotransplantation of islets and bone marrow in STZ-induced diabetic mice indicated significantly low blood glucose levels high serum insulin levels, and increased serum NGF levels. A significant increase in the number of vessels within the graft area at day 14 after transplant was observed along with improvement in graft function [218]. Thus, an important adjuvant role of transplanted BMSCs in both angiogenesis and *β*-cell regeneration has been elucidated. Cotransplantation of pancreatic islets and adipose-tissue-derived stem cells (ADSCs) too was shown to significantly prolong graft survival and insulin function of islet grafts in diabetic mice. ADSCs have angiogenic potential and anti-inflammatory properties and cotransplantation studies indicated significant revascularization (larger number of von Willebrand factor-positive cells) and marked inhibition of inflammatory cell infiltration, including CD4+ and CD8+ T cells and macrophages, in islets-ADSCs grafts [219]. Creation of a rich subcutaneous vascular network with implanted adipose-tissue-derived stromal cells and adipose tissue enhanced subcutaneous grafting of islets in diabetic mice [220]. Cografting of neural crest stem cells with pancreatic islets in alloxan-induced diabetic mice too improved insulin release and enhanced *β*-cell proliferation, resulting in increased *β*-cell mass [221]. Multipotent human MSCs possess powerful *ex vivo* expansion and EC differentiation potential, placing them at the forefront in the field of vasculature-directed cell-based therapy and transplantation. A recent study revealed that islets co-cultured with MSCs demonstrated lower ADP/ATP ratios, higher GSIS indexes and viability. Furthermore, co-cultured islets revealed higher levels of antiapoptotic signal molecules (XIAP, Bcl-xL, Bcl-2, and heat shock protein-32), increased VEGF receptor 2 and Tie-2 mRNA expression and elevated levels of phosphorylated Tie-2 and focal adhesion kinase protein [222]. Islets cultured in MSC-conditioned medium (MSC-CM) for 48 hr significantly lowered blood glucose levels and demonstrated enhanced blood vessel formation upon transplantation into STZ induced diabetic mice. Significant levels of IL-6, IL-8, VEGF-A, HGF, and TGF-*β* were detected in MSC-CM suggesting that the trophic factors secreted by human MSCs enhanced islet survival and function after transplantation. Similar improvement of islet graft morphology and function attributable in part to the promotion of graft revascularization was observed when Lewis rat islets cocultured with syngeneic MSCs were infused into the liver of STZ-diabetic syngeneic recipients or when islets were cotransplanted under the renal capsule of NOD*scid* mice with syngeneic MSCs expanded in culture [223]. Other studies have demonstrated increased mean capillary density upon co-transplantation of MSCs with pancreatic islets along with improved islet graft function indicating the beneficial effect of MSC-mediated graft vascularization [224]. In mice, islets co-transplanted with MSCs maintained a morphology closely resembling that of islets in the endogenous pancreas, both in terms of size, and of endocrine and EC distribution. Superior vascular engraftment as shown by increased EC numbers within the endocrine tissue as well as improved graft function demonstrated by normoglycemia achieved in 92% of mice indicated that MSCs profoundly influenced the remodeling process, by improving islet revascularization and maintaining islet organisation [224, 225]. In order to study the effect of MSCs on islet survival and insulin secretion under hypoxia/reoxygenation- (H/R-) induced injury conditions that are associated with islet graft dysfunction, purified rat islets cultured with or without MSCs, were exposed to hypoxia (O(2) ≤ 1%) for 8 h followed by reoxygenation for 24 and 48 h, respectively [226]. MSCs maintained a higher level of stimulation index (SI) of GSIS in islets *in vitro*, protected islets from H/R-induced injury by decreasing the apoptotic cell ratio and increasing HIF-1*α*, HO-1, and COX-2 mRNA expression, and significantly increased insulin expression following islet transplantation. This study indicated that MSCs could promote anti-apoptotic gene expression by enhancing their resistance to H/R-induced apoptosis and dysfunction providing an experimental basis for the therapeutic use of this strategy for enhancing islet function. 

## 3. Insulin-Secreting *β*-Cell Generation from Stem Cells for Replacement Therapy in T1D

Apart from the beneficial therapeutic role of stem cells in immunomodulation and promoting vasculogenesis following islet transplantation, they may also play an important role in regeneration of *β*-cells. The concept of regenerating *β*-cells from a self renewing, expandable stock of pluripotent ESCs, pancreas-derived multipotent progenitor/stem cells, extrapancreatic adult stem cells (e.g, BMSCs, neural progenitor cells, UCB-SCs, etc.) or iPSCs into large quantities of cells with an insulin-expressing phenotype *in vitro* offers an attractive alternative source for *β*-cell replacement therapy [227, 228]. While the immunosuppressive, anti-inflammatory, and angiogenic properties of MSCs are of tremendous advantage, the ability of iPSCs to generate an unlimited supply of clinically compliant, functional autologous *β*-cells provides a definitive solution to the cited limitations of islet transplantation, namely, shortage of donor pancreases and the harmful side effects of chronic immunosuppressive therapy. 

Based on sequential exposure of human ESCs to epigenetic signals that mimic *in vivo* pancreatic development, insulin-producing cells can be generated from ESCs [227, 229–231]. These differentiated cells display architectural similarity to mature primary islets, are capable of synthesizing insulin, glucagon, somatostatin, pancreatic polypeptide and ghrelin [229, 230], reverse hyperglycemia in diabetic mice, prolong graft survival, and respond successfully to glucose challenge in glucose-tolerant tests (GTT) providing definitive evidence of the ability of hESCs to serve as a renewable source of insulin-secreting *β*-cells for diabetes cell-replacement therapies. The risk of teratoma formation and tumor formation and the difficulty of purifying the differentiated progeny are major drawbacks. Multipotent BM-MSCs are plastic-adherent cells, expressing surface markers such as CD90, CD73, CD105, CD44, and CD29 that can be isolated and expanded with high efficiency in culture and can differentiate into cells of connective tissue lineages, including bone, fat, cartilage, and muscle [232]. The capacity of BM-MSCs to generate insulin-producing cells [232, 233] capable of producing and releasing insulin in a glucose-dependent manner and normalizing hyperglycemia upon transplantation into a diabetic mice [234, 235] while at the same time abrogating immune injury, altering T cell cytokine pattern toward IL-10/IL-13 production and preserving CD4+/CD8+ FOXP3+ Tregs in the periphery [236] make BM-MSCs an invaluable tool in *β*-cells replacement therapies. *In vivo *differentiation of hUCB cells into *β*-cells following transplantation into STZ-induced diabetic immunocompromised [237, 238] or NOD mice [239] indicates the potential role of these cells in *β*-cell replacement therapy. iPSCs may be derived from autologous somatic cells by ectopic expression of the transcription factors *Oct4*, *Sox2*, c-*myc*, and *Klf4* or oct3/4, sox2, nanog, lin28 [240] and are molecularly and functionally highly similar to ESCs [241], offering an important alternative to replenish *β*-cell supply [242] while simultaneously obviating immune concerns such as rejection and chronic immunosuppression. Most human iPSC lines can be induced into Pdx1-positive progenitor cells and further differentiated into pancreatic lineage cells using a stepwise induction strategy [243, 244]. Other alternative sources of *β*-cells include intrahepatic biliary epithelial cells and gall bladder epithelium [245], human neural progenitor cells [246], hepatic oval cells [247] placenta-derived multipotent stem cells [248], and adult pancreatic stem/progenitor cells [249, 250]. The common embryonic origin of liver and pancreas, similarities in their glucose-sensing systems, mutually expressed transcription factors, and the high level of developmental plasticity exhibited by adult human liver cells indicate the therapeutic potential of liver stem cells/hepatocytes as a source of pancreatic progenitor tissue. Several studies have demonstrated reprogramming of hepatocytes into function insulin-producing cells by expression of the Pdx1 or its superactive form Pdx1-VP16 fusion protein either alone or in combination with other pancreatic transcription factors using first generation, nontoxic, transiently expressed adenoviral vectors under conditions of hyperglycemia or hepatic regeneration [251–253]. However, although the potential of stem cells in the future of T1D interventional therapies is immense, the accompanying risk of mutagenesis, teratoma and tumor formation needs to be stringently addressed. For now it appears that the combination of multiple therapeutic avenues is required to achieve the dream of permanently reversing/preventing T1D.

## 4. Conclusion

Areas of current research include the development of less toxic immunosuppressive regimens, the suppression of inflammatory responses immediately following transplantation, the identification of an optimal anatomical site for islet infusion, and the possibility of encapsulating transplanted islets to protect them from the alloimmune response. The generation of Tregs with defined alloantigen specificity could provide dynamic control of rejection responses and offer a potential route to permanent graft survival without the need for life-long nonspecific immunosuppression. Regeneration of *β*-cells utilizing every kind cell from the pancreas, stem cells as well as cells from alternate sources, followed by transplantation using immunosuppressive regimens that would ensure maximal graft survival through protection from hypoxic and immune insults is an exciting alternative. Clinical islet transplantation represents a possible definitive intervention for patients with T1D and with significant inroads in the branches of stem cell therapy, immunomodulation and gene therapy, the prospect of translating these beneficial interventions into clinical applications that promote successful long-term functional islet graft survival appears within reach.
